# From Genes to Therapy in Autism Spectrum Disorder

**DOI:** 10.3390/genes13081377

**Published:** 2022-08-01

**Authors:** Jacob A. S. Vorstman, Christine M. Freitag, Antonio M. Persico

**Affiliations:** 1 Department of Psychiatry, University of Toronto, Toronto, ON M5S 1A1, Canada; 2 Program in Genetics and Genome Biology, Research Institute and Autism Research Unit, The Hospital for Sick Children, Toronto, ON M5G 0A4, Canada; 3 Department of Child and Adolescent Psychiatry, Psychosomatics and Psychotherapy, Autism Research and Intervention Center of Excellence, University Hospital Frankfurt, Goethe-Universität, 60528 Frankfurt am Main, Germany; c.freitag@em.uni-frankfurt.de; 4 Department of Biomedical, Metabolic and Neural Sciences, University di Modena e Reggio Emilia, 41225 Modena, Italy; antonio.persico@unimore.it

In recent years, findings from genetic and other biological studies are starting to reveal the role of various molecular mechanisms that contribute to the etiology of ASD. These growing insights from fundamental research have broadened the emphasis in the field from gene discovery alone to burgeoning efforts to explore the clinical translation of this new knowledge [1,2,3]. A key premise for these efforts is the idea that molecular mechanisms involved in ASD may be targeted by pharmacological strategies [4,5]. This shift from gene discovery to the development of novel therapeutic strategies is the theme for a Special Issue of this journal, bringing together eight invited manuscripts, each providing a unique angle on this exciting topic. Here, we will review and contextualize these contributions against a background of recent literature and discuss the emerging prospects for the field of ASD.

While knowledge derived from genetic studies may help inform research funding and guide efforts towards novel therapeutic strategies, the fact that the same knowledge can already provide directly actionable insights for the clinical management of individual patients, is currently still largely overlooked [6]. These clinical insights are not only relevant with respect to various aspects related to reproductive counseling (e.g., probability of recurrence); in some cases, it can also directly inform therapeutic management of the individual patient. For example, Dyar et al., discuss how dosage of various psychotropic medications may require adjustment in those patients with Phelan–McDermid Syndrome with 22q13.3 deletions including the gene *CYP2D6* [7]. Clinically actionable information derived from genetic testing is also center stage in the discussion of three individual patients with different genetic conditions, reported by Butler et al., These examples, drawn from clinical practice, illustrate how such knowledge can already inform clinicians about preventive monitoring, the need for additional medical examinations or for surveillance for specific conditions associated with the genetic variant as well as guide the choice of psychotropic medications [8,9].

Genetic test results are by no means the only information source which can have direct clinical relevance; together with other observations, such as brain electrophysiological measures, this information can be used to preselect patients for certain types of treatments, as was recently elegantly argued [10]. This is the approach taken by the collaborative BRAINMODEL project, reported by Geertjens et al., which focuses on patients with specific genetic variants and subsequently aims to integrate patient-specific biomarker data obtained through different data modalities. These include in vitro synaptic (network) activity in patient-iPSC-derived neurons and electroencephalography, obtained separately in each enrolled patient with the sole objective to elucidate the best therapeutic strategy for this individual person [11].

The number of genes with solid evidence for a relation to ASD has increased considerably over the past years and continues to increase with time [12]. The partial overlap with genes relevant to developmental delay and intellectual disability adds another layer of complexity [13,14]. Whether genes exist that predominantly increase the probability of developing ASD, and not or to a lesser degree the probability of intellectual disability, is a subject of debate and may require more extensively phenotyped datasets to answer [15,16]. The extreme scenario, with every single genetic etiology representing a single pathophysiology of ASD, clinical translation would be facing the formidable challenge of having to design roughly as many treatments as identifiable genetic etiologies. However, findings are pointing towards a strong convergence of genetic heterogeneity in a limited number of pathways [17], which can impact neurodevelopment either directly (“neuronal” and “synaptic” genes) or indirectly by mainly deranging epigenetic mechanisms (“transcriptional regulation” and “chromatin remodelling” genes) [18,19].

One of these convergent pathways, indicated by the involvement of several genes in which pathogenic variants can increase the probability of ASD, is synaptic function: this pathway has been discussed in several contributions present in this Special Issue. For example, *Vasic* et al. [20]. discuss the connections between observations in humans and those derived from murine studies of genes involved in so-called “signalopathies”, including *PTEN*, *TSC1*, *TSC2* and *NF1*, and how their point of convergence is abnormal synaptic function [20]. A potential role of disrupted synaptic function is also inferred from the association with ASD of genes such as *SHANK3*—the gene central to Phelan–McDermid Syndrome-, *NRXN1* and *CNTNAP2*. In humans, these, and other genes involved in synaptic transmission are not only associated with ASD behaviors; they are also related to measurable differences in brain development. For example, Bieneck et al., show that the physiological cortical thinning in individuals with ASD during adolescence was decreased compared to typical controls; this possibly indicates abnormalities in the process of synaptic pruning. Interestingly, the observed differences in cortical thinning correlated with synaptic genes [21]. These findings echo previous observations, indicating an association between synaptic genes and differences in cortical thickness in individuals with ASD [22,23]. Bieneck et al., reported altered cortical thinning predominantly in fronto-temporal brain regions and the cingulate cortex in individuals with ASD [21]. In addition, these developmental changes were associated with severity of repetitive behaviors, while the most affected brain regions were enriched for genes involved in synaptic function, corroborating earlier studies indicating the involvement of synaptic genes in cortical thickness [22]. These observations cannot be unconditionally generalized, given the complexity of neurodevelopmental synaptic processes. For example, *SHANK3* haploinsufficiency in Phelan–McDermid syndrome is associated with prominent white matter damage, especially evident by diffusion tensor imaging (DTI) in the uncinate tract, the inferior fronto-occipital fasciculus, and other long association fiber tracts, while gray matter volumetric abnormalities appear more subtle [24,25]. Nonetheless, such observations, establishing the link between molecular pathways, markers of brain development, and behavior, underscore the clinical relevance of genetic findings [22].

Indeed, proposed over a decade ago [26,27,28], the biological convergence of different genetic variants on synaptic function is no longer merely theoretical. A concrete clinical implementation of this knowledge is one of the premises of the previously mentioned collaborative BRAINMODEL project which identifies one of its target groups by the presence of pathogenic variants affecting synaptic function [11]. Large-scale projects, such as EU-AIMS, follow the same rationale of stratifying individuals with ASD for clinical trials based on underlying pathophysiology [29].

The study of the behavioral phenotypes in mouse models of these genes generates relevant information that cannot be easily obtained in human carriers of these variants, feeding essential information into the efforts towards novel pharmacological interventions [30]. Illustrating this principle, their observations in *Shank3*, *Nrxn1* and *Cntnap2* knockout mice indicate that many of the phenotypic abnormalities are detectable early in life. This is not only consistent with the emergence of symptoms early in life in individuals with ASD; it also coincides chronologically with increased brain expression of these, and other, developmental genes [30]. Indeed, the earlier the expression, the greater the clinical intersection between ASD and developmental delay [19]. Similar findings were highlighted by Vasic et al., providing a rationale for the importance of timing interventions early in development [20]. These and other studies suggest that many of the synaptic genes found to be associated with ASD are expressed prenatally and throughout early development, spatially and temporally coinciding with synaptic organization in brain regions relevant for ASD. Together with the emergence of ASD symptoms in humans early in life and with the physiological timing of synaptogenesis during prenatal and early postnatal stages in human brain development [31], these observations suggest that early in life may be the optimal window for treatment interventions. These notions provide a useful framework to interpret the somewhat disappointing results of the initial trials examining efficacy of novel compounds in genetically selected human target populations, such as Fragile-X [32,33,34]. On the one hand, human patients were probably treated beyond the “point-of-no-return” represented by the end of critical periods for plastic recovery in imbalanced neural circuits; on the other hand, compared to murine species, the human brain seemingly has much more stringent molecular constraints and vastly greater complexity, both neuroanatomically and in terms of transcriptional control [35]. Taken together, the promising findings in murine studies and the disappointing experiences of the first human trials provide a strong rationale to consider optimal therapeutic windows when examining psychopharmacological compounds going forward [35]. In this context, it is important to note that the phenotypic impact of pathogenic variants is highly variable, both in terms of severity (i.e., variable expressivity) and of the nature of the phenotype (pleiotropy) [36,37]. Variable penetrance and pleiotropy pose a significant challenge from the perspective of treatment, in particular when interventions are expected to be implemented very early in life. The probability for developing ASD is, for most pathogenic variants, not 100%, but more typically in the range of 20–75% [38]. Consequently, efforts are needed to improve accuracy of the prediction of developmental trajectories in the individual patient. In this regard, the genomic context in which rare high impact variants occur likely modifies risk in the individual carrier, as was recently suggested by findings in individuals with the 22q11.2 deletion [39]. A recently initiated international consortium, Genes to Mental Health (G2MH) examines how additional genomic (and environmental) factors can be used to refine prediction of neurodevelopmental outcomes such as ASD in carriers of rare genetic variants with high impact [40]. Despite these efforts, DNA variation alone is unlikely to explain the majority of phenotypic variance, since epigenetic variation is as efficient as deletions in down-regulating expression of critical ASD genes [41] and the modulation of ASD gene promoter methylation is emerging as a significant cause of association between ASD and many common SNPs [42].

Early phenotypic manifestations may also contribute to improving our ability to foresee atypical development towards ASD early in life [43,44]. In this context, the increased interest in manifestations of altered sensory processing as a core phenotype of ASD is particularly enticing. Of particular interest are their emergence during the first year of life [45], and their correlation with the other autistic symptom domains [46]. These observations underscore their potential as early biomarker of ASD [47] and as clinical endpoint measure in trials, such as proposed by the BRAINMODEL project [11]. The rationale for such novel approaches is further strengthened by the observation of sensory abnormalities in mouse models of various genetic variants associated with ASD [20,30,48]. Disruptions of synaptic function, such as those leading to disturbances in the excitation-inhibition homeostasis [49] or to loss of synaptic scaling [50], may well underlie abnormal sensory processing, providing further evidence for the link between certain pathogenic genetic variants and this component of the ASD phenotype.

The manner in which genetic knowledge currently informs the development of novel treatment strategies for ASD is no longer limited to the selection of novel or repurposed compounds acting on the putatively involved molecular pathways. Genetics-informed strategies now also increasingly include approaches aimed at restoring the direct consequences of the mutation on the availability of a functional gene product. Weuring et al., review the pre-clinical studies of such technologies, including those who do not include edits in the genome (“transient”), mostly relying on interference with RNA. These transient methods can be contrasted to those that edit the genome (“permanent”), which rely on the replacement of genes by the integration of cDNA in the subjects genome, or editing of a genomic gene sequence [51]. Encouraged by the observed clinical efficacy of Nusinersen (Spinraza), an antisense oligonucleotide for the treatment of spinal musical atrophy [52], similar strategies are currently examined for over 10 genetic conditions associated with ASD [51].

Several articles in this Special Issue describe the contribution that patient-derived iPSCs and derived neuronal models can give to our understanding of the pathophysiology of ASD, especially in reference to known genetic disorders [11,20]. Furthermore, being able to test the therapeutic effects of pharmacological agents in these models provides a great opportunity to bypass the limitations imposed by species-specific epigenetic control and functional responses when using primary cultures from murine models. The possibility to assess these cellular models at multiple levels of analysis, encompassing structural dynamics, dendritic spine and synapse formation, electrophysiological network activity recorded using microelectrode arrays, to single-cell transcriptomics and so on, confers unprecedented capabilities to collect pathophysiologically and pharmacologically relevant data directly from human cells. Yet, no single model can in and by itself summarize and encompass all levels of systemic complexity present in a neurodevelopmental disorder, underscoring the importance of viewing this information as complementary to data derived from animal models, which are also presented and discussed by several groups in this Special Issue [11,20]. 2-D patient-derived neural networks and even 3-D “minibrains” [53] still represent an oversimplified model of the complex interactions between different cellular types and neurotransmissions in the Central Nervous System (CNS). As an example, iPSCs from patients with different forms of ASD in the context of specific genetic disorder, including Phelan–McDermid syndrome, provide convergent evidence of decreased excitatory synaptic transmission [11]. However, animal models carrying FMR1, CNTNAP2, 16p11.2, TSC2 or SHANK3 haploinsufficiency all display a more profound compensatory decrease in inhibitory neurotransmission as compared to excitation level, so that the final outcome consists of an E > I imbalance even if excitation is decreased, as shown by iPSC models [54]. In addition, animal models provide unique information on developmental trajectories, the timing of critical periods, the correlation between specific genetic disruptions and neurochemical, neurophysiological and behavioral correlates. These considerations explain why a balanced view between iPSC and animal models of ASD has been sought in this Special Issue, under the assumption that together these complementary approaches can provide maximum pre-clinical support to the experimental clinical pharmacology of neurodevelopmental disorders.

In her contribution, D’Gama discusses a challenging chapter in ASD genetics, namely, the role of genetic variants which arise in somatic cells of the proband (de novo) or of his/her parents (transmitted). Somatic mutations have been estimated to contribute to ASD risk in at least 3–5% of simplex families, but this is likely an underestimation [55]. Compared to germline mutations, disease mechanisms involving somatic mutations are particularly challenging for at least two major reasons. First, they are highly tissue- and cell-type specific. The accessibility of genomic DNA carrying somatic mutations is a major hurdle for brain-related disorders, such as ASD. As commented by D’Gama, deep sequencing of cell-free DNA extracted from cerebrospinal fluid (CSF) raises hope but is currently still under scrutiny [56]. Tissue source and biomaterial collection strategy may well raise difficulties in applying this knowledge into clinical practice. Secondly, the epilepsy literature provides evidence that as little as 1% of mutated neurons is sufficient to yield drug-resistant epilepsy both in humans and in animal models [57]. Conceivably, the CNS may be more sensitive to the presence of cells carrying functional somatic mutations, as compared to parenchymal organs such as kidney and liver. Relatively few mutated cells may be enough to generate a bioelectrical imbalance in neural networks or to derange connectivity during development. This great CNS sensitivity to somatic mutations, paired with the absence of germline genetic variants able to explain ASD by single-hit in the majority of patients, raise interest in studying somatic mutations and finding ways to translate this knowledge into clinical practice despite these hurdles and caveats.

## Conclusions

Several themes reverberate throughout the different contributions to this Special Issue. When reflecting on how growing insights in the genetic underpinnings of ASD can be translated to therapeutic advances for this population, one paradox inevitably transpires, i.e., that of extensive genetic heterogeneity of ASD on the one hand, and the convergence into a limited number of biological pathways. Of the different biological pathways, synaptic function is increasingly scrutinized as a possible mechanism amenable to targeted pharmacological interventions. Findings in murine models suggest the importance of the early timing of intervention. Despite its greater resilience to genetic haploinsufficiency, greater sensitivity to targeted pharmacological interventions, and obvious limitations in brain circuitry, the developing mouse brain retains validity in many areas which can provide a framework for interpreting the somewhat disappointing trial results obtained thus far in human ASD.

A second theme that emerges—echoing similar developments in the entire field of medicine—is the importance of precision health approaches. Such approaches hinge upon our ability to stratify the population of individuals with ASD into subgroups based on shared pathophysiology. Within groups of patients at risk for neurodevelopmental disorders based on shared carriership of a high impact genetic variant, refinement of individual risk is required to overcome the challenge of variable expressivity and pleiotropy invariably associated with genetic risk. Individualized treatments can in some instances be already pursued today, provided genetic results are analyzed also with this aim in mind [7,8], and clinicians have received sufficient training in their interpretation [58]. Further momentum will hopefully be provided by the translation of iPSC-based approaches into individualized pharmacological treatments [11,20]. This final step in personalizing treatment will usually imply targeted psychopharmacological interventions, but may also go as far as applying genome editing, at least in some monogenic forms of ASD [51].

A third leitmotif is the direct link between genetic variants and specific symptom domains, such as stereotyped and repetitive behaviors [59,60] or EEG abnormalities/comorbid epilepsy due to E > I imbalance [11]. This link may be a frequent mediator of the broader association between these genetic variants and ASD altogether. Several contributions in this Special Issue have underscored the importance of altered sensory processing and reactivity both in animals models of ASD and in human studies [11,30]. This domain has taken the front stage in recent years after its explicit recognition as a diagnostic criterion for ASD in the DSM 5. The relevance is at least twofold: on the one hand, this domain may be more directly linkable to specific and traceable neurobiological abnormalities in comparison to other ASD domains falling within the broader realm of social cognition; on the other hand, abnormal sensory processing is increasingly emerging as a relevant source of maladaptive behaviors with measurable impact on the quality of life of individuals with ASD and their families.

In summary, the past decade has provided a solid rationale for advancing the field towards precision medicine treatments in ASD, based on the ongoing discovery of genetic contributions and their convergence into a limited number of biological pathways. During the current decade, we may be witnessing the actual first implemented steps in this direction, as elegantly illustrated by the collected articles in this Special Issue.
